# Differential neural mechanisms for early and late prediction error detection

**DOI:** 10.1038/srep24350

**Published:** 2016-04-15

**Authors:** Rahim Malekshahi, Anil Seth, Amalia Papanikolaou, Zenon Mathews, Niels Birbaumer, Paul F. M. J. Verschure, Andrea Caria

**Affiliations:** 1 Institut für Medizinische Psychologie und Verhaltensneurobiologie, Universität Tübingen, Tübingen, Germany; 2 Graduate Training Centre of Neuroscience, International Max Planck Research School, Tübingen, Germany; 3 Sackler Centre for Consciousness Science and School of Informatics, University of Sussex, Brighton, UK; 4 Max Planck Institute for Biological Cybernetics, Tübingen, Germany; 5 SPECS, Universitat Pompeu Fabra, Barcelona, Spain; 6 Department of Psychology and Cognitive Science, University of Trento, Rovereto, Italy

## Abstract

Emerging evidence indicates that prediction, instantiated at different perceptual levels, facilitate visual processing and enable prompt and appropriate reactions. Until now, the mechanisms underlying the effect of predictive coding at different stages of visual processing have still remained unclear. Here, we aimed to investigate early and late processing of spatial prediction violation by performing combined recordings of saccadic eye movements and fast event-related fMRI during a continuous visual detection task. Psychophysical reverse correlation analysis revealed that the degree of mismatch between current perceptual input and prior expectations is mainly processed at late rather than early stage, which is instead responsible for fast but general prediction error detection. Furthermore, our results suggest that conscious late detection of deviant stimuli is elicited by the assessment of prediction error’s extent more than by prediction error *per se*. Functional MRI and functional connectivity data analyses indicated that higher-level brain systems interactions modulate conscious detection of prediction error through top-down processes for the analysis of its representational content, and possibly regulate subsequent adaptation of predictive models. Overall, our experimental paradigm allowed to dissect explicit from implicit behavioral and neural responses to deviant stimuli in terms of their reliance on predictive models.

Predictive processing refers to the brain mechanisms that infer the flow of sensory information based on learned regularities of inputs data^1^,^2^,^3^,^4^. Emerging evidence indicates that prediction, instantiated at different perceptual levels, facilitate visual processing^5^,^6^,^7^ and enable prompt and appropriate reactions^8^. Redundancy reduction and neural coding efficiency achieved through predictive processing represent spatio-temporal functions of the classical receptive field^7^,^9^,^10^ as well as of higher level aspects of visual processing^11^.

According to predictive coding models cortical feedback connections mediate top-down predictive information initiated by stimulus presentation, whereas feed-forward signals convey bottom-up prediction error signals^1^,^7^,^12^,^13^. Discrepancies between higher-level predictive models and lower-level inputs induce adaptive changes of generative models so as to cancel error of prediction and to generate perceptual inference 4,14,15,16,17. Perception is then postulated to result from a minimization of sensory prediction error.

Expected regular information, as confirmation of formulated predictions, is promptly and accurately processed^18^. Sequential regularities are automatically encoded by the visual sensory system^19^,^20^,^21^,^22^,^23^. On the other hand, prediction error^24^,^25^,^26^ as indicator of changes in the environment or unsuccessful learning should be preferentially encoded^27^,^28^,^29^,^30^. Analysis of prediction error associated with ‘deviant’ stimuli generates dynamic changes in neural representations to enable perception as well as fine-tuning of internal models of the environment^31^,^32^,^33^,^34^,^35^. Generative models are thus constantly updated in a hierarchical fashion so that prediction in a lower level is subordinated by prediction in an upper level, and sensory information represented at the lowest level triggers adjustment and optimization of expectations at higher levels^36^.

To date, it remains unclear how predictive coding influences different stages of visual processing. Models of visual cognition and empirical evidence have suggested two-stages of information processing^37^,^38^,^39^,^40^: an early, implicit, stage for fast processing and evaluation of visual stimuli and a second slower stage implying selective attention for accurate detection of specific properties, and necessary for visual awareness. Contrasting evidence still exists about how prediction regulates early and late visual processing. Several investigations demonstrated that prediction error signal is processed implicitly^41^,^42^,^43^,^44^,^45^ and outside the focus of attention^46^,^47^,^48^. On the other hand, it has been shown that violation of prediction, being highly relevant for behavior, gains preferential conscious access^49^,^50^,^51^. However, some other studies suggested that early implicit preattentive signals precede conscious detection of unexpected deviant stimuli^52^.

Here, with the intent to clarify the mechanisms underlying early and late visual processing of prediction violation we performed combined recordings of saccadic eye movements and fast event-related functional magnetic resonance imaging during a continuous visual detection task. Participants were required to make a button press response whenever they detected a moving item that was suddenly displaced with respect to its current linear trajectory. Our task exploited previous results showing that visual motion induces a spatial forward prediction of future patterns of sensory input along the motion path^53^,^54^,^55^,^56^.

As predictive processing is postulated to be a top-down process we hypothesized that spatial prediction would affect detection of deviant stimuli more markedly at late rather than at early stage. Early prediction error detection, which is essential for triggering fast behavioral response, would then allow a general analysis of the type of violation, whereas higher level processing would subserve specific assessment of prediction error’s properties.

Early detection of deviant stimuli was based on express saccades^57^,^58^,^59^ whereas late detection was based on regular saccades and on explicit button press responses.

To characterize behavioral differences between early and late responses to deviant stimuli we employed psychophysical reverse correlation, a powerful method that permits to uncover participants’ internal representations and decision strategies during visual tasks^60^, including motion perception^61^.

At neural level, we expected increasing response latencies to deviant stimuli to be associated with strengthened functional interactions among hierarchical brain systems, as indication of increased exchanges of top-down and bottom-up information. Specifically, fronto-parietal and thalamo-cortical activity was supposed to characterize late prediction error detection, as these networks are known to support the generation and modification of appropriate forward models^15^,^62^,^63^,^64^,^65^,^66^,^67^, whereas early processing of spatially deviant stimuli was predicted to engage activity in the inferior parietal cortex, a region frequently associated with visual detection^68^,^69^. Interactions among brain regions were investigated using functional connectivity data analysis.

## Results

**Figure 1:**
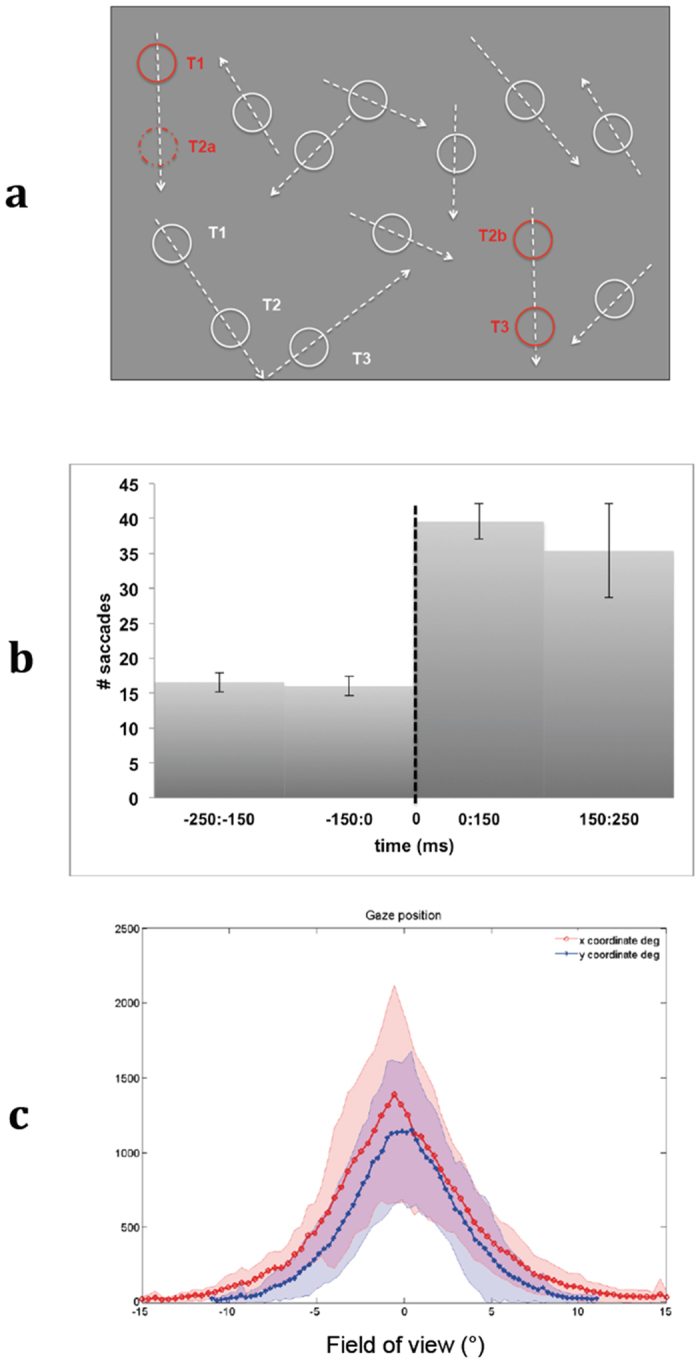
(**a)** Visual scenes consisted of multiple identical non-filled white circles (n = 10) moving along linear trajectories on a gray background and bouncing off display boundaries. Participants were required to detect a visual item (depicted in red for illustrative purpose only) moving along a predictable trajectory that from time to time was spatially displaced with variable changes in amplitude and orientation (inter-displacement time ranged between 2000 and 4000 ms). A 0° amplitude corresponded to a displacement in the same trajectory of the moving item. The randomly selected displaced item (e.g. red dotted circle at time T2a) continued a linear motion (T2b - > T3) with the same direction and speed prior to displacement (T1 - > T2a) but in a different position. (**b)** Average number of express and regular saccades ( ± SD) towards visual target within 250 ms time windows before and after displacement (time = 0). (**c)** Distribution of gaze position (number of times over each display position) during our visual detection task. The red and blue curves represents how many times ( ± SD) the gaze was oriented towards each x and y coordinate respectively. Participants reported having mainly focused on the central part of the scene when debriefed; sometimes they referred being captured by moving stimuli and having performed singular smooth pursuit eye movements but they reported not having tracked multiple moving items.

In our study we employed psychophysical reverse-correlation analysis to determine what decision strategies mediated participants’ performance during detection of spatially deviant stimuli (Fig. 1a). In particular, this approach permitted to investigate how the probability of detection of a displacement with a given amplitude and orientation with respect to the moving direction varies for early (express saccades) and late responses (either regular saccades and button presses). This was achieved by comparing at different perceptual stages an elliptical region - referred to as the *psychophysical kernel* - depicting detectable and undetectable stimuli as function of the degree of deviancy. This region is characterized by specific parameters such as *area, eccentricity, shift* and *orientation*.

### Behavioral data

**Figure 2:**
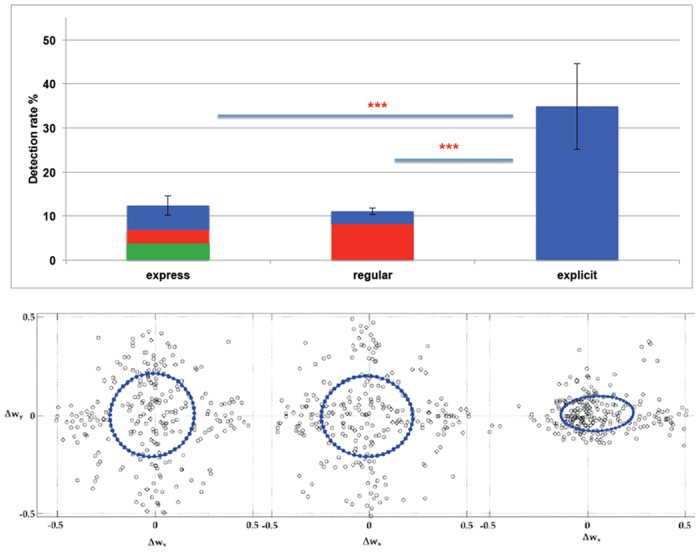
Top: Detection rate at different perceptual levels. The average detection rate was 34.87% ± 9.70 (mean ± SD) for explicit detection (blue bar), 11.06% ± 0.78 for regular saccades (red bar, mutually exclusive), and 12.37% ± 2.17 for express saccades (green bar, mutually exclusive). Sometimes, express saccades were not mutually exclusive: 3.03% of all displacements were detected with express saccades followed by regular saccades, 5.5% with express saccades followed by explicit detection, and 2.84% with regular saccades followed by explicit detection. *** denotes a significant difference in detection rate (p = 0.001). **Bottom**: The three ellipses represent the psychophysical density kernels for express (left) and regular saccades (middle), and explicit detection (right). The ellipses are depicted in the polar coordinate system; considering the maximum displacement amplitude and pixel per angle, the range of angular displacement Δw ± 0.5 represents the min and max values of the kernel’s axes. The density kernels represent how the probability of detection of a displacement, with a given amplitude and orientation with respect to the moving direction, varies at different perceptual levels. The blue dotted circle represents the covariance ellipse of the 2D Gaussian distribution, also referred to as the psychophysical kernel. An item falling inside the ellipse has higher probability not to be detected and vice versa for items outside the ellipse. The unit of displacement is degrees of field of view, which are normalized with respect to movement direction and speed. Considering the maximum displacement amplitude and pixel per angle, the values range ± 0.5 represent the min and max values for the axis of the kernel (smoothing made displacements near to the center - small displacements - appearing close to each other and not easily trackable, whereas displacements far from the center - large displacements - are more easily distinguishable along the eight target directions). The analysis of psychophysical kernel properties indicated that with increasing response latencies detection to deviant stimuli changed in relation to the degree of deviancy of the expected trajectory. Late detection required large deviation, whereas early detection, that occurred less frequently, was equal for large and small deviating stimuli.

Behavioral results showed an average detection rate of 34.87% ± 9.70 (mean ± SD) for explicit responses, 11.06% ± 0.78 for regular saccades, and 12.37% ± 2.17 for express saccades (Fig. 2 top). Sometimes, express saccades were not mutually exclusive: 3.03% of all displacements were detected with express saccades followed by regular saccades, 5.5% with express saccades followed by explicit detection, and 2.84% with regular saccades followed by explicit detection.

A significant difference of detection rate was measured between explicit detection and express saccades (t_11_ = 11.18: p < 0.001), and between explicit detection and regular saccades (t_11_ = 10.43: p < 0.001); no significant difference in detection rate was measured between regular saccades and express saccades (t_11_ = 1.86: p = 0.078). Participants reported having mainly focused on the central part of the scene when debriefed; this strategy was also confirmed by eye movement distribution (Fig. 1c). Sometimes they referred being captured by moving stimuli and having performed singular smooth pursuit eye movements but they reported not having tracked multiple moving items.

In addition, we observed a significant higher number of express saccades towards displacement locations as compared to express saccades towards non-displacement locations (two tailed t-test t_11_ = 10.33: p < 0.0001), indicating that unpredictable deviant stimuli indeed increased the number of express saccades^59^,^70^.

### Psychophysical reverse correlation

Psychophysical reverse correlation was employed to compute a two-dimensional probability distribution for detection and non-detection densities (Fig. 2 bottom). The covariance ellipse of the Gaussian distribution, the psychophysical kernel, represents the area where displaced items have more probability not to be detected, normalized to the direction of travel.

The ANOVA showed that the main factor type of response (express and regular saccades, and explicit detection) was significant for area (F(2, 11) = 38.20, p < 0.001, ηp2 = 0.810), eccentricity (F(2, 11) = 15.27, p < 0.001, ηp2 = 0.743), shift (F(2, 11) = 12.42, p < 0.001, ηp2 = 0.900) but not for orientation (F(2, 11) = 2.28, p = 0.130, ηp2 = 0.519). Post-hoc t-tests revealed that area was larger for express saccades (t_11_ = 7.34, p < 0.001), and regular saccades (t_11_ = 5.71, p < 0.001) with respect to explicit detection, whereas eccentricity and shift were significantly higher for explicit detection than for express saccades (eccentricity: t_11_ = 4.20, p = 0.002; shift: t_11_ = 4.87, p = 0.001), and regular saccades (eccentricity: t_11_ = 5.02, p = 0.001; shift: t_11_ = 5.88, p < 0.001). A significant difference in eccentricity was also observed between express and regular saccades (t_11_ = 2.39, p = 0.044). No significant differences were observed between express saccades and express saccades followed by button press for any of the psychophysical kernel properties.

The analysis of psychophysical kernel properties indicated that late detection of deviant stimuli, either regular saccades or explicit detection, significantly differed from detection with either express saccades in relation to the degree of deviancy, and so to the predicted trajectory. In particular, increasing eccentricity for late responses, explicit detection as compared to regular saccades, and regular saccades as compared to express saccades, indicated that from fast to slow responses the number of detectable trials was increasingly higher for items displaced along perpendicular or negative direction (more deviating from predicted trajectory) as compared to those displaced along positive direction (less deviating from predicted trajectory) (Fig. 2 bottom). In short, late detection required large deviation, whereas early detection, that occurred less frequently, was equal for large and small deviating stimuli.

### fMRI data

**Figure 3:**
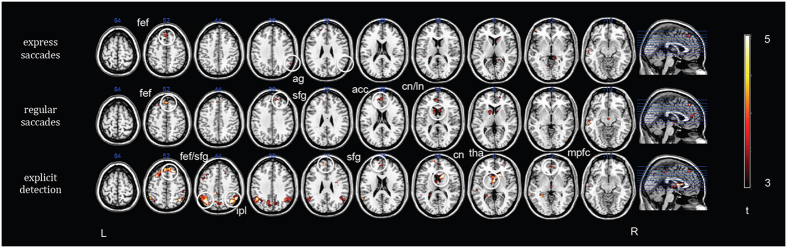
SPM t-maps of brain activations comparing detected > undetected trials for express saccades, regular saccades, and explicit detection. Increasing response latencies – regular saccades and explicit detection – were associated with larger involvement of frontal and prefrontal regions as well of subcortical structures such as thalamus and caudate nucleus than for express saccades. Fef = frontal eye fields; ag = angular gyrus; sfg = superior frontal gyrus; acc = anterior cingulate cortex; cn = caudate nucleus; ln; lentiform nucleus; ipl; inferior parietal lobule; tha = thalamus; mpfc = medial prefrontal cortex; pc = precuneus; mfg = middle frontal gyrus; L = left; R = right. SPM(t) maps were thresholded at p < 0.05 (cluster-wise false discovery rate (FDR) correction for multiple comparisons); the cluster-forming threshold was set to p < 0.005.

Brain areas with increased blood oxygenation level-dependent (BOLD) response resulting from separates contrasts comparing express and regular saccades and explicit detection to undetected trials are listed in Table 1 and depicted in Fig. 3. Prediction error detection with express saccades compared to undetected trials was associated with activations in the right angular gyrus and frontal eye fields (FEF). Detection with regular saccades compared to non-detected trials activated the FEF, the right superior frontal gyrus (BA9), the left middle temporal gyrus (BA21) and the anterior cingulate cortex, and at subcortical level the caudate nucleus and the lentiform nucleus. Explicit detection as compared to undetected trials was associated with a fronto-parietal network distributed bilaterally that included the frontal eye fields (BA8), the medial frontal gyrus (BA 9, 10), the inferior parietal lobule and supramarginal gyrus (BA39, 40), the orbitofrontal cortex (BA10), the cuneus and precuneus (BA 7) and the anterior cingulate cortex (BA32); at subcortical level activations were observed in the caudate nucleus and thalamus. Among detected trials, detection with regular saccades as compared to detection with express saccades revealed activations in the left precuneus (BA7), and left and right anterior cingulate cortex (BA32) (Table 1). Explicit detection as compared to detection with express saccades revealed activations in the right superior and middle frontal gyrus (BA 10), left precuneus (BA7) and right anterior cingulate cortex (BA32) (Table 1). As no significant activations were observed in motor and premotor regions we excluded residual activity associated with preparation and execution of hand-related motor behavior.

**Figure 4:**
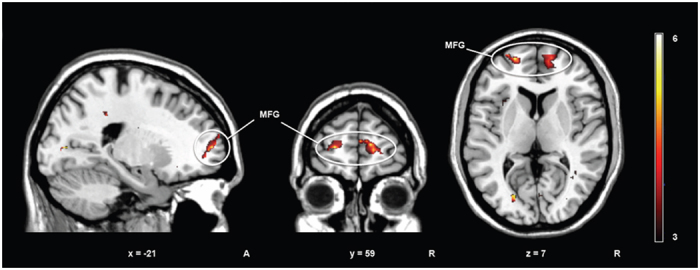
Whole brain activity correlating with *eccentricity.* Eccentricity is a psychophysical kernel property that describes visual detection as ratio between stimuli with large and small deviation from the expected trajectory. Within regions where activity was increased during explicit detection, the most significant clusters corresponded to the left and right middle frontal gyrus (BA10, −27, 47, 7; 30, 41, 13).

When testing for whole brain activity correlating with *eccentricity* we observed that, within regions where activity was increased during explicit detection, the most significant clusters corresponded to the left and right middle frontal gyrus (BA10, −27, 47, 7; 30, 41, 13; Fig. 4).

In summary, our results showed that the right inferior parietal lobule (supramarginal/angular gyrus) is involved in both early and late prediction error detection. Increasing response latencies – regular saccades and explicit detection – were associated with larger involvement of frontal and prefrontal regions as well of subcortical structures such as thalamus and caudate nucleus than for express saccades. Moreover, regression analysis indicated that the medial prefrontal cortex is associated with the assessment of the degree of deviancy from the expected trajectory.

### Functional connectivity analysis

The modulatory effect of the inferior parietal cortex (supramarginal/angular gyrus) on the remaining brain areas was assessed comparing explicit detection with express saccades. This region was selected on the basis of our fMRI results showing activity during both early and late responses, and on its known role for the detection of deviant stimuli^68^,^69^.

A spherical region (6 mm radius) centered on the right inferior parietal cortex (MNI coordinates x, y, z = 60, −58, 28) was then selected as ‘seed’ region. The coordinates of the center of the sphere were identified based on the peak maximum obtained from conjunction analysis (Minimum Statistic compared to the Conjunction Null (MS/CN))^71^ testing for the conjunction of the contrasts explicit detection *versus* undetected trials and detection with express saccades *versus* undetected trials.

Psychophysiological interactions (PPI) analysis during explicit detection as compared to express saccades showed that the right inferior parietal lobule was positively coupled with the anterior cingulate cortex (12, 35, 1), the right middle frontal gyrus (BA10) (12, 65, 19), and the left middle temporal gyrus (−48,−25,−14).

## Discussion

In this study we investigated at behavioral and at neural level the influence of spatial prediction on visual detection of experimentally manipulated deviant stimuli at different perceptual stages. To this aim we combined recordings of saccadic eye movements and fast event-related fMRI while participants performed a continuous visual task implying detection of stimuli violating the expected linear trajectory.

Our behavioral results indicated that visual motion-induced spatial prediction differentially affects early and late visual detection of spatially deviant stimuli. By depicting the distributions of both detected and undetected deviant stimuli we observed that the extent of prediction violation influenced stimulus detection at late but not at early stage. The comparison of psychophysical kernel properties between early and late responses revealed significant differences for *eccentricity, shift and area*. Specifically, explicit detection was associated with a smaller area with respect to detection with express saccades, indicating that the number of undetectable trials for explicit detection was smaller (and correspondingly the number of detectable trials was larger) than for detection with express saccades. *Eccentricity* was greater for explicit detection than detection with express saccades, and for regular saccades with respect to express saccades, indicating that for slower responses detection of displacement in the direction of item’s movement (expected linear trajectory) was more difficult than in other directions. On the contrary, fast saccadic detection (express saccades) was quite independent of stimulus deviation as evidenced by *eccentricity* closer to zero. Overall, we observed a smaller number of saccades as compare to button presses. Saccadic responses were in principle not necessary for detection of the displacement; in fact, as some subjects reported, the optimal strategy for successful completion of the task was to fixate the center of the screen and to detect displacements peripherally, where sensitivity for motion cues is still maintained^72^.

These results are in line with previous studies showing that early saccadic responses do not integrate spatio-temporal information, and target displacements can pass unnoticed because the precise location is not yet transferred^73^. Accordingly, the influence of the degree of deviancy on late but not on fast detection might be ascribable to the slower processing of spatio-temporal information, which implies that early comparison of current inputs with top-down prediction cannot consider such information.

In a previous study we observed a similar bias in detection of deviant stimuli under high cognitive load condition but it was unclear whether this was related to the increased cognitive load or to predictive mechanisms^56^. Here we provide indications of a general prediction-based mechanism that differentially impacts early and late processing of prediction violation.

On the basis of our results we argue that prediction error elicited by deviant stimuli might signal different information at early (implicit) and late (explicit) level. It has been shown that prediction error response induced by deviant stimuli can convey information about identity of the mismatch with expected information^74^. As the degree of violation did not influence early saccadic detection we assume that this fast response might be mainly regulated by a general ‘surprise’ effect associated with violation of prediction, whereas late detection would be additionally modulated by the degree of violation, that is, the extent of mismatch with prior information. However, the measured effect might also partially result from a reduced ‘surprise’ effect associated with our task, as all stimuli to be detected were deviant.

Nevertheless, our results are in line with previous studies showing that relevant prediction error conveying information about critical contingencies in the environment needs to be further processed in order to adapt our internal models^75^,^76^.

The relevance, or salience, of prediction error permits to detect a stimulus and to discard it when it is not relevant to the current predictive model^77^,^78^. In our experiment, displacement’s orientation and amplitude, which might be inherently associated with its relevance, would have then called for a higher-level assessment.

Furthermore, as the majority of explicitly detected stimuli were those with large deviation from the expected trajectory we speculate that deviant stimuli have to be highly deviant in order to be sufficiently salient to gain conscious access.

Currently, two main hypotheses have been formulated regarding the role of prediction on conscious processing. On one hand, it has been proposed that conscious access occurs when predictive models are verified against sensory inputs so that prediction errors are minimized^2^,^21^,^79^,^80^,^81^. According to this hypothesis confirmation of prediction would be critical for consciousness. On the other hand, it has been suggested that conscious access depends on the mismatches between predictions and sensory input^49^,^50^,^51^, and thus errors of prediction would trigger conscious perception. Our results suggest that the mismatch alone is not sufficient and that its extent would be as well important for conscious detection. Our findings would then call for a refinement of the general hypothesis proposing that unexpected stimuli would gain preferential conscious access because of their inherent increased relevance. However, our task was different from those used in recent studies investigating how prediction affects detection^21^,^80^ inasmuch as only deviant stimuli were our perceptual target. Additional evidence on the role of stimulus’ relevance on conscious visual processing is thus required.

### Neural correlates of early and late deviant stimuli detection

Our fMRI results showed that early processing of deviant stimuli (detection with express saccades *versus* undetected targets) engaged activation of a portion of the inferior parietal lobule, the right angular gyrus. Previous studies reported early event-related potentials components associated with detection of sequential deviants, as well as rare visual targets in the oddball task, located in the posterior occipito-temporal regions^68^,^82^. The right angular gyrus is also part of the temporo-parietal-junction, which is active during detection of novel events and attentional reorienting^24^,^83^,^84^. Activation in the occipital–parietal network was previously associated with express saccades as compared to regular saccades using a simple visually guided saccade task (saccadic response to the appearance of a peripheral visual target)^85^.

While express saccades are likely controlled by reflexive mechanisms (sensory driven), regular saccades, which are characterized by longer latency, might also reflect volitional (internally driven) commands^86^,^87^, and thus possibly representing an intermediate level of processing towards the explicit response. Accordingly, regular saccades were associated with increased activity in brain regions involved in higher-order oculomotor control^86^,^88^,^89^,^90^. Specifically, detection with regular saccades as compared to undetected stimuli included activations in the ACC, the superior frontal gyrus (FEF) (BA8, 9), and in the lentiform and caudate nuclei, the latter representing a central input of the oculomotor basal ganglia.

On the other hand, explicit button press responses, which were more influenced by predictive information of stimulus trajectory, were associated with brain activity of frontal and prefrontal regions, and parietal cortex. Explicit detection as compared to undetected trials showed activations of the middle and superior frontal gyrus (BA 8, 9), medial and orbital frontal gyri (BA 10), bilateral inferior parietal lobule (BA 40), including supramarginal and angular gyri, precuneus (BA7), anterior cingulate gyrus (BA 32), caudate nucleus and thalamus.

Previous studies investigating violation of expectation identified specific functional networks associated with detection of deviant stimuli^63^,^68^,^91^. Conscious detection of violation of auditory stimuli regularity as compared to non-deviant stimuli was associated with distributed activity in a fronto-parietal network including the bilateral dorsolateral prefrontal and anterior cingulate cortex^63^,^68^,^91^. Differences in brain activations were also observed during detection of visual stimuli violating a relational structure in comparison with detection of a predefined stimulus using a task that did not require regularities to be inferred^63^. While a large involvement of right premotor and prefrontal areas was associated with detection of sequential deviants, mainly bilateral activations in parietal (inferior parietal lobule) and temporal (inferior and middle temporal gyrus) cortices were related to target detection. In our study, as the target to be detected was a deviant stimulus, brain activations are similar to those previously reported for the detection of both deviant and target stimuli. However, brain activations related to the ‘surprise’ effect of unexpected stimuli should be marginal as both detected and undetected stimuli were deviant and unpredictable.

During explicit detection we observed activity in the dorsolateral prefrontal cortex (BA 9), which was previously associated with preparation of forthcoming actions and with the monitoring of information in working memory^92^,^93^, in the prefrontal cortex (BA 10) and in the left inferior parietal lobule (BA 40), which were instead reported during target detection only^63^. We did not see significant changes in BOLD signal in the premotor regions, which have been associated with sequential processing possibly reflecting instantiation of forward predictive models. Such mechanism would be plausibly present during both detection and non-detection of deviant stimuli.

Explicit detection also activated the right inferior parietal lobule, which was shown to be involved with detection of deviants irrespective of the kind of expectations being violated (e.g. position, rhythm, and object identity). We conjecture that this region might have a general role in prediction error detection^63^,^68^,^82^. The activation of the right inferior parietal cortex might be necessary but not sufficient to assess the relevance of the degree of deviancy; additional involvement of frontal and prefrontal regions would be instead indispensable^94^,^95^. We indeed observed activations of parietal regions - precuneus (BA7) – and frontal areas - anterior cingulate cortex (BA 32) - during late responses, either explicit detection or regular saccades as compared to express saccades, and additionally of the medial prefrontal cortex (BA 10,11) during explicit detection as compared to express saccades.

Importantly, whole brain regression of BOLD activity within explicit detection against *eccentricity* - which describes stimulus detectability in relation to its predictability/unpredictability - also showed activation of medial prefrontal cortex (BA10). This result further supports the role of this region in shaping explicit detection based on predictive information^66^,^67^.

Functional connectivity analysis of explicit detection as compared to express saccades revealed that the right inferior parietal cortex was positively coupled with ACC and with the medial prefrontal cortex (BA10), suggesting that prediction error evaluation might be supported by an exchange of information between frontal/prefrontal and parietal regions, which are anatomically and functionally interconnected.

Previous studies identified the orbitofrontal cortex in the context of prediction error detection^96^,^97^ and proposed that generation and modification of appropriate forward models might be subserved by this region and by the medial prefrontal cortex^15^,^62^. A number of fMRI studies also demonstrated that the prefrontal cortex is critical for establishing forward models based on sequential regularities^63^,^64^,^65^. The involvement of the medial prefrontal cortex during explicit detection would then potentially indicate additional analysis of the salience of the mismatch between current input and prior expectations^66^,^67^.

Finally, our results are in line with previous studies showing the critical role of ACC in encoding prediction error between stimulus expectation and outcome^98^,^99^. In particular, the dorsal ACC would encode the so called absolute prediction error, a Bayesian surprise signal important for detecting and adapting to drastic, unexpected changes in the environment, as well as the signed prediction error, representing the valence of error of prediction^100^,^101^,^102^.

## Conclusions

Our behavioral and functional imaging data suggested that implicit detection of prediction error enable first fast but partial comparison of bottom-up sensory input with top-down predictive information^15^, whereas a slower processing would permit a more comprehensive assessment of the type of mismatch between actual and predicted information. Overall, our experimental paradigm allowed to dissect explicit from implicit behavioral and neural responses to deviant stimuli in terms of their reliance on predictive models. Moreover, our results indicated that conscious detection of deviant stimuli is elicited by the assessment of prediction error’s extent rather than by prediction error *per se*. Finally, based on our findings we postulate that explicit rather than implicit behavior might be critical for triggering brain processes tuning generative forward models with sensory feedback.

## Methods

### Participants

Twelve volunteers with normal or corrected-to-normal vision (5 women; aged 20–33 years; mean = 28.41, SD = 3.98) participated in the study. All participants had no history of neurological or psychiatric disorders including substance abuse/dependence or psychotropic medications. Participants were carefully instructed not to move, while relaxing and breathing regularly in order to avoid potential artifacts due to physiological changes. Before scanning, a test session was performed outside the scanner to allow participants to become familiar with the task and instructions. Written informed consent was obtained from all participants before being involved in the study. This study was approved by the Ethics Committee of the Medical Faculty of the University of Tübingen according to the Declaration of Helsinki. The methods carried out in this work are in accordance with the approved guidelines.

### Stimuli and experimental procedure

Participants underwent a continuous visual task during which they were required to detect a visual item, moving along a predictable trajectory, that from time to time was spatially displaced with variable changes in amplitude and orientation (deviant stimuli)^56^. Visual scenes consisted of multiple identical non-filled white circles (n = 10) moving along linear trajectories on a gray background and bouncing off display boundaries (Fig. 1a). The radius of all circular items was 1.0° of visual angle. All stimuli moved with a constant speed of 18°/s; a slight change in speed was induced ( ± 0.001°/s) at boundary bounces to produce minimally different linear paths. Every t seconds a randomly selected moving item was displaced from its linear trajectory and then continued a linear motion with the same direction and speed prior to displacement but in a different position. Inter-displacement time t was drawn randomly from a uniform distribution ([2000,4000] ms). We specifically manipulated the angles of displacement with respect to moving direction, which were counterbalanced and selected pseudo-randomly from 8 possible directions: 0°, ± 45°, ± 90°, ± 135°, 180°. The amplitudes of displacement ranged between 0.5° and 8° of visual angle with 0.5° step. A 0° amplitude corresponded to a displacement in the same trajectory of the moving item. To ensure linear motion before and after displacements and reduced potential bias due to visibility effects, items with a distance from a boundary less than two times the planned displacement radius were excluded. Participants were not instructed to fixate or to saccade, but to look freely anywhere on the screen. Presentation of multiple moving items reduced automatic singular movement smooth-pursuit^103^, and induced participants to adopt different strategies than tracking moving objects. In this way we aimed to maintain the effect of motion predictability, which has been shown to be significantly reduced when participants perform multiple objects tracking tasks^104^.

Early responses were measured based on express saccades - latency between 25 and 150 ms^57^,^58^,^59^ - which represent an implicit stimulus-driven eye movement that can be reflexively elicited by the appearance of a new item^59^, and can be predictive of the expected target location^70^. Previous studies also showed that uncertainty about location and time of onset of a visual target – which characterizes our experimental protocol - increased the occurrence of express saccades^58^. Late responses were based on regular saccades - latency between 150 and 250 ms^57^,^58^,^59^ - and on explicit detection followed by button press response. Regular saccades cannot be considered a pure explicit response because of their relatively short latencies, nor a pure implicit response, as it has been shown that in some circumstances they can be volitionally controlled^86^,^87^. Thus, regular saccades represented an intermediate level of processing towards the explicit response.

Participants underwent two runs of 160 trials each during which they had to press a button (with the index finger of the right hand) whenever they noticed a displaced item. Stimuli were displayed through VisuaStim goggles (Resonance Technology Company, Northridge, CA; 30° horizontal field of view with a spatial resolution of 800 × 600 pixels) incorporating an eye-tracking system. Stimulus presentation was implemented using Psychopy [1.73.06]^105^. Button press responses and trajectories of moving stimuli were logged time-synchronized with eye tracking data.

### Eye tracking

An infrared eye tracker mounted on the goggles was used to record the eye movements (Resonance Technology Company, Northridge, CA). Eye movements were sampled at 60 Hz. A calibration phase was performed before the experiment for each subject. Calibration error was below 0.5° of visual angle for nine calibration points. After appropriate preprocessing (e.g. blink detection), an algorithm based on eye movement acceleration was used to detect the beginning of saccades^106^,^107^. Two main criteria had to be satisfied to determine a saccade towards a displaced item: eye movement oriented within ± 22.5° around a straight line between eyes and displaced item, and acceleration above an adaptive threshold computed as one standard deviation from the mean of a 2 s time-window preceding the item’s displacement^107^. A significant difference between the number of saccades towards visual target within 250 ms time windows before and after displacements confirmed the validity of our approach (two tailed t-test t_11_ = 8.77, p < 0.001, Fig. 1b). All data were analyzed using Matlab R2013b (The Mathworks, Natick, MA) and custom scripts.

### Behavioral data analysis

#### Psychophysical reverse-correlation

Behavioral data were analyzed using psychophysical reverse-correlation. This method allowed us to determine what decision strategies mediated participants’ performance in our visual detection task. Psychophysical reverse-correlation has been successfully employed to explore the relationships between a *high-dimensional* variable (e.g. an image - in our case the distribution of displacements of moving stimuli) and a *categorical* variable (two-choice decision or neural spiking^60^,^108^, - in our case ‘detected’ and ‘undetected’ trials).

In our analysis each displacement was plotted as point in the direction-normalized coordinate system, where the positive x-axis represents the linear movement direction of the moving item. Detected trials were binned into three categories according to the type of detection^56^: detection with express saccades and regular saccades, and explicit detection followed by button press. Data analysis mainly focused on mutually exclusive detection trials in order to clearly characterize early and late visual detection mechanisms.

Data were first normalized with respect to movement direction and speed. Average detection and non-detection densities were computed for each perceptual level; data interpolation yielded a two-dimensional probability distribution for detection and non-detection densities separately. The difference between the two probability distributions (detected and undetected) was computed. We performed 1000 times sampling of the resulted density distribution and calculated the covariance of the sampled points. Data were then fitted with a 2D Gaussian distribution. The covariance ellipse of this Gaussian distribution is referred to as the *psychophysical kernel*. The kernel (centered at the location where an item would have been if it had not been displaced) represents the probability of a displacement with a given amplitude and orientation to be detected. An item falling inside the ellipse has higher probability not to be detected and vice versa for items outside the ellipse. Four different properties of the psychophysical kernel were computed separately for each type of response (express and regular saccades, explicit response): *area*, *eccentricity*, *shift* and *orientation*. In case of no differences in displacement detection with respect to amplitude and direction the kernel is circular (*eccentricity* = 0) and centered at the origin (*shift* = 0). Differences in the four psychophysical kernel properties among conditions were analyzed using four independent ANOVAs with type of response (express and regular saccades, and explicit detection followed by button press) as within-subjects factor and consecutive post-hoc t-tests. Data were checked for normality and homoscedasticity. Normality was confirmed by the skewness and kurtosis of the distributions. Homoscedasticity was confirmed by the Mauchly test, which was not significant for the distribution of *eccentricity*, *shift*, and *orientation*, but significant for *area*; in the latter case the Greenhouse-Geisser correction was considered.

### fMRI data acquisition

A fast event-related functional MRI paradigm of two consecutive runs was adopted. During fMRI runs participants performed a displacement detection task. Functional images were acquired using a 3.0 T MR scanner, with a standard 12-channel head coil (Siemens TIM Trio Magnetom, Erlangen, Germany available at the Max Plank Institute, Tuebingen, Germany). A standard echo-planar imaging (EPI) sequence was used (repetition time TR = 1.5 s, matrix size = 64 × 64, effective echo time TE = 30 ms, flip angle α = 70°, bandwidth = 1.905 kHz/pixel). Twenty-three axially oriented slices (voxel size = 3 × 3 × 3.3 mm^3^, slice gap = 0.57 mm) were acquired. For superposition of functional maps upon brain anatomy a high-resolution T1-weighted structural scan of the whole brain was collected from each subject (MPRAGE, matrix size = 512 × 512, 176 partitions, voxel size = 1 × 1 × 1 mm^3^, TR = 1950 ms, TE = 2.26 ms, TI = 900 ms, α = 9°). In order to minimize head movements, two foam cushions were positioned around participant’s head.

### fMRI data analysis

Functional imaging data were analyzed using SPM 8 (Wellcome Department of Cognitive Neurology, London, UK). All functional images were first motion corrected and realigned. The high-resolution T1 image was then co-registered to the mean image of the EPI series for each participant. Segmentation parameters were used to normalize the functional scans to a standard Montreal Neurological Institute template. Normalized images were spatially smoothed with a 10mm full-width half-maximum Gaussian kernel. Low frequency drifts were removed using a high-pass filter with 128 s cut off. After functional data preprocessing, a general linear model was adopted to perform first level statistical analysis. For each participant, an analytic design matrix was constructed using the following type of events as regressors: express saccades, regular saccades, explicit response, button press and undetected trials. Only mutually exclusive detection trials were included in the analysis; multiple response trials were combined in an additional regressor. Conditions were modeled with a canonical hemodynamic response. We considered the onset time of item displacement instead of button press to reduce potential effect of motor response on brain activations related to explicit detection. Consequently, onset time of item displacement was also used to define express and regular saccades. We then included temporal derivatives into our model to take into account time variability of response with respect to item’s displacement. Additional regressors for button press were also included to cancel out residual hand movement-related variance.

For each participant, contrast images of detected versus undetected trials were created for express and regular saccades, and explicit responses. The contrast explicit detection *versu*s detection with express saccades, and detection with regular saccades *versus* detection with express saccades were also considered. Because of the different number of trials for each type of response weighted contrasts were considered when comparing conditions.

In our experimental task participants were required to continuously attend the visual scene and be prepared to press a button whenever a displaced item was noticed. Thus, possible confounding activity related to motor preparation and attention should be marginal when contrasting different conditions.

Contrast images were then entered into a second-level (random-effects) analysis to allow population-level inferences. One sample t-tests on the contrast images imported from the first-level analysis were performed to assess group effects across all participants. Additionally, we performed a regression analysis of images related to the contrast explicit detection *versus* undetected trials, where psychophysical kernel parameters resulting from psychophysical reverse correlation analysis were modeled as covariates. We considered *eccentricity* among other parameters as it uniquely describes visual detection as ratio between stimuli with large and small deviation from the expected trajectory. The resulting SPM(t) maps were thresholded at p < 0.05 (cluster-wise false discovery rate (FDR) correction for multiple comparisons^109^,^110^. The cluster-forming threshold was set to p < 0.005.

### Functional connectivity analysis

Psychophysiological interaction analysis (PPI toolbox available in SPM8)^111^ was employed to assess changes in functional connectivity during late explicit as compared to early prediction error detection. PPI is a model-free functional connectivity method that determines whether a given region, the seed/ source, ‘predicts’ the activity in other brain regions as a function of a task/context specific factor. Kim and colleagues using a biologically plausible neural model showed that PPI analysis reflects the underlying changes in neural interactions as results were similar to those based on integrated synaptic activity^112^.

For a selected ‘seed’ region, the first eigenvector of a predefined area is then used for calculating the context-dependent changes in interregional covariance. To this aim the difference in regression coefficients between the neuroimaging signals of this region and the rest of the brain is tested^113^. PPI results were corrected for multiple comparisons at cluster level (p corrected < 0.05; cluster size estimated at p uncorrected < 0.005).

## Additional Information

**How to cite this article**: Malekshahi, R. *et al.* Differential neural mechanisms for early and late prediction error detection. *Sci. Rep.* **6**, 24350; doi: 10.1038/srep24350 (2016).

## Figures and Tables

**Table 1:** Brain activity associated with express saccades, regular saccades and explicit detection, and resulting from the comparisons regular saccades > express saccades and explicit detection > express saccades.

Location	Side	Coordinates (MNI)	K_E_	BA	t value
**Express saccades (implicit detection)**					
Angular gyrus	R	60, –58, 28	11	39	4.30
Superior frontal gyrus	L	−3, 35, 49	15	8	3.98
Medial frontal gyrus	L	−3, 38, 43	–	8	3.44
**Regular saccades**					
Middle temporal gyrus	L	−63, −25, −8	35	21	4.21
Superior frontal gyrus	L	−6, 38, 49	41	8	5.63
Caudate nucleus	R	12, 11, 13	28		5.60
Superior frontal gyrus	R	15, 53, 40	20	9	5.46
Lentiform nucleus	L	−12, 2, 1	74		4.20
ACC	L	−6, 38, 19	83	32	4.61
**Explicit detection**					
Middle frontal gyrus	R	27, 32, 49	244	8	13.74
Superior frontal gyrus	L	−6, 29, 52		8	10.46
Caudate nucleus	R	15, 17, 10	201		10.91
Caudate nucleus	R	12, 17, 4	–		10.07
Caudate nucleus	L	−12, −1, 16	–		5.79
Superior frontal gyrus	L	−6, 53, 28	67	9	7.91
Medial frontal gyrus	L	−9, 62, 16	–	10	7.18
Medial frontal gyrus	R	6, 50, 19	–	9	4.83
Inferior parietal lobule	L	−57, −49, 46	293	–	7.39
Angular gyrus	L	−57, −58, 25		39	6.46
Supramarginal Gyrus	L	−57, −58, 29	–	40	6.46
Inferior parietal lobule	R	42, –61, 43	253	40	7.87
Superior parietal lobule	R	39, –67, 49	–	7	7.79
Supramarginal gyrus	R	57, –58, 31	–	40	7.29
Medial frontal gyrus	R	6, 47, 1	58	32	6.01
Orbital frontal cortex	L	−3, 53, −5	–	10	5.49
Superior medial frontal	R	3, 56, 10	–	10	5.02
Precuneus	L	−6, −64, 40	41	7	5.92
Cuneus	L	−6, −76, 31	–	7	5.27
Thalamus	L	−8, −4, 10			5.05
Anterior cingulate cortex	R	3, 47, 1		32	5.67
**Regular saccades > express saccades**					
Precuneus	L	−12, −70, 40	65	11	5.22
Anterior cingulate cortex	R	12, 41, 7	26	32	4.82
Anterior cingulate cortex	L	−9, 32, 7	10	32	4.17
**Explicit detection > express saccades**					
Middle frontal gyrus	R	27, 50, −2	24	11	10.95
Superior frontal gyrus	R	27, 59, 7	–	10	5.85
Precuneus	L	−9, −64, 43	65	7	5.09
Anterior cingulate cortex	R	6, 47, 7	26	32	5.61

Activations were thresholded at p < 0.05 (cluster-wise false discovery rate (FDR) correction for multiple comparisons); the cluster-forming threshold was set to p < 0.005.
