# Corrigendum: Differential neural mechanisms for early and late prediction error detection

**DOI:** 10.1038/srep46590

**Published:** 2017-04-11

**Authors:** Rahim Malekshahi, Anil Seth, Amalia Papanikolaou, Zenon Mathews, Niels Birbaumer, Paul F. M. J. Verschure, Andrea Caria

Scientific Reports 6: Article number: 2435010.1038/srep24350; published online: 04 15 2016; updated: 04 11 2017

In this Article, an additional affiliation for Paul F.M.J. Verschure was omitted. The correct affiliations for Paul F.M.J. Verschure are listed below:

SPECS, Universitat Pompeu Fabra, Barcelona, Spain.

ICREA - Institució Catalana de Recerca i Estudis Avançats, 08018 Barcelona, Spain.

